# Visualizing the Potential Impairment of Polymyxin B to Central Nervous System Through MR Susceptibility-Weighted Imaging

**DOI:** 10.3389/fphar.2021.784864

**Published:** 2021-12-02

**Authors:** Ni Zhang, Lichong Zhu, Qiuhong Ouyang, Saisai Yue, Yichun Huang, Shuang Qu, Runwei Li, Yuanyuan Qiao, Man Xu, Fangfei He, Bin Zhao, Lai Wei, Xiaoai Wu, Peisen Zhang

**Affiliations:** ^1^ Department of Psychiatry, and Department of Nuclear Medicine, West China Hospital, Sichuan University, Chengdu, China; ^2^ College of Life Science and Technology, Beijing University of Chemical Technology, Beijing, China; ^3^ Xinxiang Key Laboratory of Forensic Toxicology, School of Forensic Medicine, Xinxiang Medical University, Xinxiang, China; ^4^ Department of Rehabilitation Medicine, School of Medicine, Guangzhou First People’s Hospital, South China University of Technology, Guangzhou, China

**Keywords:** polymyxin B, neurotoxicity, Fe_3_O_4_ nanoparticles, in vivo, susceptibility-weighted imaging

## Abstract

Polymyxin B (PMB) exert bactericidal effects on the cell wall of Gram-negative bacteria, leading to changes in the permeability of the cytoplasmic membrane and resulting in cell death, which is sensitive to the multi-resistant Gram-negative bacteria. However, the severe toxicity and adverse side effects largely hamper the clinical application of PMB. Although the molecular pathology of PMB neurotoxicity has been adequately studied at the cellular and molecular level. However, the impact of PMB on the physiological states of central nervous system *in vivo* may be quite different from that *in vitro*, which need to be further studied. Therefore, in the current study, the biocompatible ultra-uniform Fe_3_O_4_ nanoparticles were employed for noninvasively *in vivo* visualizing the potential impairment of PMB to the central nervous system. Systematic studies clearly reveal that the prepared Fe_3_O_4_ nanoparticles can serve as an appropriate magnetic resonance contrast agent with high transverse relaxivity and outstanding biosafety, which thus enables the following *in vivo* susceptibility-weighted imaging (SWI) studies on the PMB-treated mice models. As a result, it is first found that the blood-brain barrier (BBB) of mice may be impaired by successive PMB administration, displaying by the discrete punctate SWI signals distributed asymmetrically across brain regions in brain parenchyma. This result may pave a noninvasive approach for in-depth studies of PMB medication strategy, monitoring the BBB changes during PMB treatment, and even assessing the risk after PMB successive medication in multidrug-resistant Gram-negative bacterial infected patients from the perspective of medical imaging.

## Introduction

The emergence of multi-resistant Gram-negative bacteria is a major public health issue worldwide, the lack of new antibiotics led to the revival of Polymyxin B (PMB), an ancient cationic cyclic polypeptide antibiotic (Falagas et al., 2005; Zavascki et al., 2007). PMB exert bactericidal effects on the cell wall of Gram-negative bacteria, leading to changes in the permeability of the cytoplasmic membrane and resulting in cell death (Falagas and Kasiakou, 2006; Wahby et al., 2010). However, the parenteral use of these drugs was abandoned in most countries about 20 years ago, except for the treatment of patients with cystic fibrosis. Because of the common and severe reports of nephrotoxicity and neurotoxicity, the most common adverse effects of mucormycin therapy are nephrotoxicity and neurotoxicity (Velkov et al., 2018; Gallardo-Godoy et al., 2019). Nephrotoxicity consists primarily of acute tubular necrosis, as evidenced by reduced creatinine clearance and elevated serum urea and creatinine levels (Temboot et al., 2020). Neurotoxicity is associated with dizziness, weakness, abnormal facial, and peripheral sensation, vertigo, visual disturbances, confusion, ataxia, and neuromuscular blockade, which can lead to respiratory failure or apnea (Xie et al., 2020). The incidence of PMB-related neurotoxicity reported in the early literature was 7%, with sensory abnormalities being the main neurotoxic adverse event (Koch-Weser et al., 1970). Related literature reports PMB neurotoxicity characterized by perioral sensory abnormalities, ataxia, or both. Monitoring has found that patient’s signs and symptoms begin in the first few days of PMB treatment and diminish or resolve as treatment continues or the dose decreases Reported neurotoxicity includes disturbances such as dizziness (lightheadedness), altered sensation (e.g., dizziness) (numbness and sensory abnormalities affecting mainly the face), nausea, vomiting, muscle weakness, and peripheral neuropathy (Phe et al., 2014; Liu et al., 2021). More severe neurological reactions may also occur. These include confusion, coma, psychosis, convulsions, and ataxia. The appearance of these more severe neurotoxic reactions has been associated with high doses of mucin (>5 mg/kg/d) and renal damage (Bosso et al., 1991; Tuon et al., 2014).

The ability of PMB to modify or disrupt lipid membranes may help explain the toxic vulnerability of high-fat neurons (Chai et al., 2020; Fu et al., 2020). Its neurotoxicity is thought to be caused by the direct interaction of PMB with neurons, and the interaction of PMB with neurons causes dose-dependent neurotoxicity. PMB may inhibit the action of acetylcholine at the neuromuscular junction, prolong depolarization, deplete calcium, and induce histamine release (Vanhaeverbeek et al., 1974; Kelesidis and Falagas, 2015). Mild neurological symptoms of PMB usually subside after rapid discontinuation of the drug. Immediate discontinuation of PMB and other neurotoxic drugs is also the first-line approach in the presence of neuromuscular blockade (Hou et al., 2020). If apnea is present, further treatment includes mechanical respiratory support. Intravenous calcium and cholinesterase inhibitors, such as neostigmine and edrobamate, have led to conflicting results. Hemodialysis is only indicated for patients with coexisting acute renal failure (Falagas and Kasiakou, 2006; Khondker et al., 2017).

Although the molecular pathology of PMB neurotoxicity has been adequately studied at the cellular and molecular level. However, the growth status of neuron cells cultured *in vitro* is quite different from that *in vivo* (LoVerso et al., 2015; Dauth et al., 2017). Obviously, the *in vivo* researches can reflect the pathological information of the central nervous system more truly and reliably. Non-invasive research approaches provide a feasible strategy to study the nervous systems *in vivo*, which will not stimulate the nerve cells to change their growth state. Medical imaging is one of the most important means of non-invasive studies (Yang et al., 2017; Zhang et al., 2019; Zhang et al., 2020; Zhao et al., 2021). Especially, magnetic resonance imaging (MRI), one of the clinically compatible imaging modalities, have led to improved accuracy in the detection and characterization of central nervous system diseases, which can provide images of anatomical structure of the brain with high spatial resolution for studying pathologic foci (Iima and Le Bihan, 2016; Fan, 2021; Zhang et al., 2021).

Among the multiple MRI sequences, susceptibility-weighted imaging (SWI) technique is a novel imaging method that maximizes the sensitivity to susceptibility effects by combining a long-TE, high-resolution, fully flow-compensated, gradient-echo sequence with filtered phase information in each voxel to display the susceptibility difference between different tissues (Sehgal et al., 2006), which is especially sensitive to the blood products and has been proven to better track the microhemorrhages in brain in comparison with the conventional MRI sequences, i.e., *T*_1_-weighter imaging or *T*_2_-weigted imaging. Based on the imaging mechanism of this MRI modality, magnetic iron oxide (Fe_3_O_4_) nanoparticles with high saturation magnetization can serve as the best SWI contrast agents, which have been approved with a few others being at different stages of clinical trials (Abakumov et al., 2015; Gao et al., 2016; Wang et al., 2018; Song et al., 2020). Through intravenous injection, the Fe_3_O_4_ nanoparticles can sharply darken the SWI signal of the blood stream, which can definitely amplify the contrast between the brain tissues and the blood products, and depict the tiny microhemorrhage sites. Therefore, in the current work, the potential impairments of PMB to the brain was studied through SWI. Ultra-uniform Fe_3_O_4_ nanoparticles were carefully prepared and chosen as the contrast agents. The mice were intravenously medicated with PMB once a day for 3 successive days and subjected to the SWI upon the enhancement with the Fe_3_O_4_ nanoparticles. Careful imaging studies in combination with histochemical analysis of brain tissues were carried out for showing the potential impairments of the PMB to the brain of the mice. It was for the first time observed that the PMB may destroy the blood-brain barrier (BBB) of the mice after the continues medication, which reveal the impact of PMB on brain *in vivo*, and thus can provide a guideline for the clinical application of PMB, well highlighting the current studies.

## Results and Discussions

### Construction and Characterization of Fe_3_O_4_ Nanoparticles

**FIGURE 1:**
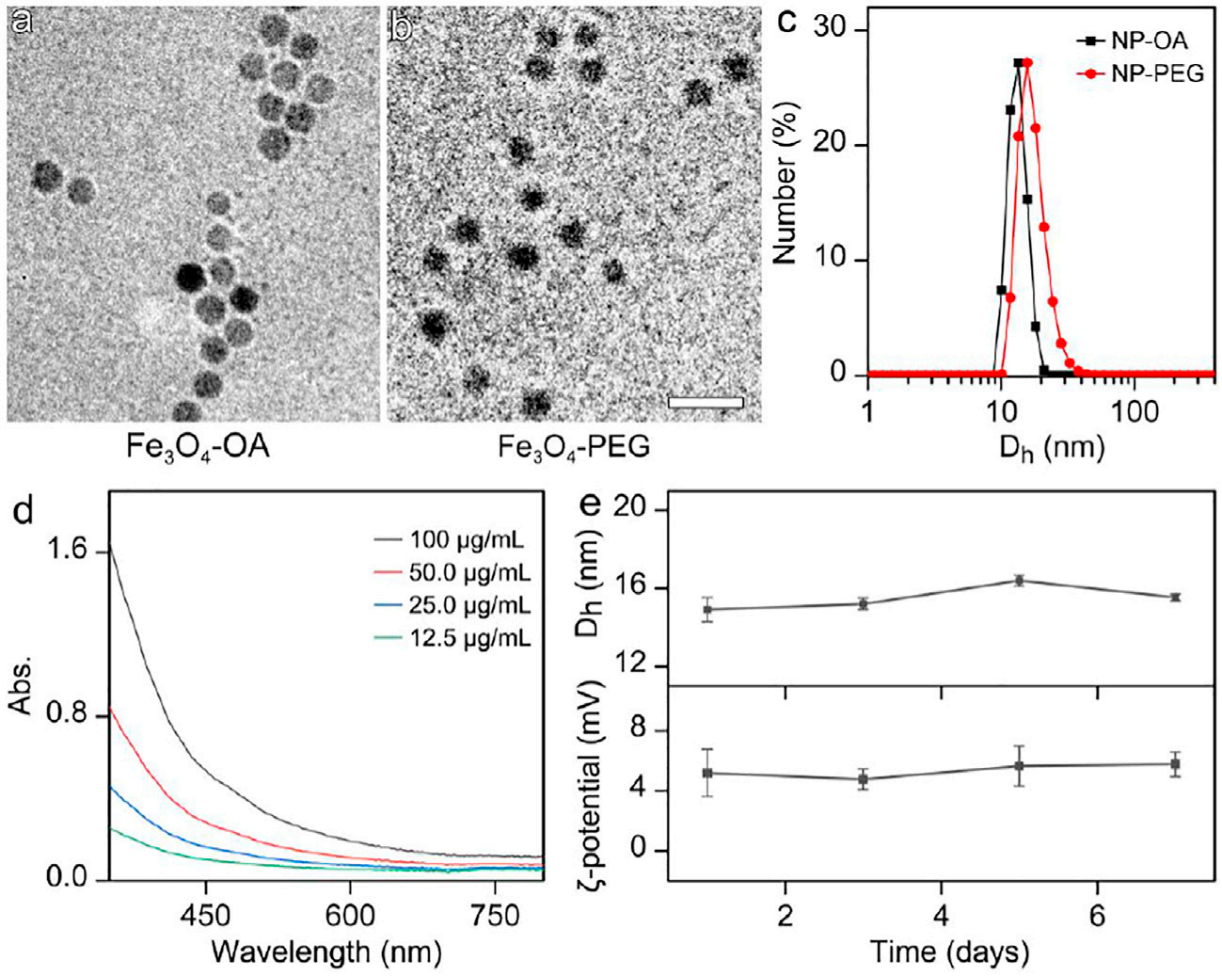
TEM images of **(a)** OA-capped Fe_3_O_4_ nanoparticles (Fe_3_O_4_-OA) and **(b)** aqueous soluble Fe_3_O_4_ nanoparticles after ligand exchange (Fe_3_O_4_-PEG). (The embedded scale bars correspond to 30 nm) **(c)** D_h_ distribution profiles of the NP-OA nanoparticles in cyclohexane solution and NP-PEG nanoparticles in water solution. **(d)** The UV-Vis absorption spectrum of aqueous solution of PEGyated Fe_3_O_4_ nanoparticles. **(e)** The temporal evolutions of D_h_ and zeta potential of the nanoparticles in water, for showing the colloidal stability of particles.

In brief, Fe_3_O_4_ nanoparticles were prepared by a high temperature approach through the thermal decomposition method. Oleic acid (OA) was chosen as both particle surface capping agent and co-solvent used together with 1-octadecene. The particle size shown in Figure 1A and size distribution shown in Supplementary Figure S1 in Supplementary Material reveal that the Fe_3_O_4_ particles were successfully synthesized with the average size of 11.3 ± 1.0 nm and a narrow size distribution. To render the Fe_3_O_4_ nanoparticles stabilized by OA water-soluble, asymmetric polyethylene glycols (PEGs) carrying a methoxyl group at one end and two phosphate groups at the other end, denoted as CH_3_O-PEG-dp, respectively, were used to replace the OA ligand of the as prepared Fe_3_O_4_ nanoparticles based on the fact that the phosphate group has a higher binding affinity to Fe^3+^ than the carboxyl group from OA. The TEM image of water soluble Fe_3_O_4_ nanoparticles were shown in Figure 1B, the particle morphology and careful statistical studies (Supplementary Figure S2) reveal that the ligand exchange did not alter the size or the size distribution profiles of the OA-capped Fe_3_O_4_ nanoparticles.

The impact of the above ligand exchange on the properties of the Fe_3_O_4_ nanoparticles was further investigated with dynamic light scattering (DLS). As shown in Figure 1C, the OA-capped Fe_3_O_4_ nanoparticles in cyclohexane present single scattering peak locating at 13.2 nm, before converting into the aqueous phase. After ligand exchange, the hydrodynamic diameter (D_h_) of Fe_3_O_4_ particles stabilized by CH_3_O-PEG-dp in water apparently increased, reaching 16.2 nm. The DLS result on the one hand suggests that PEG-phosphate can effectively replace oleate ligand. On the other hand, the size distribution profiles remain nearly unchanged in comparison with those of the hydrophobic counterparts, suggesting the surface modification occurred without undesired aggregation.

The Ultraviolet−visible absorption spectrum of aqueous solution of Fe_3_O_4_ nanoparticles was also carried out. As shown in Figure 1D, the Fe_3_O_4_ nanoparticles exhibited a broad featureless absorption covering almost the entire visible region. However, the Fe^3+^ concentration dependent absorbance spectra perfectly followed the Lambert-Beer law with the correlation coefficient at any designated single wavelength, and no significant baseline drift was observed (Supplementary Figure S3), indicating the excellent aqueous stability of the particles.

The colloidal stability of Fe_3_O_4_ nanoparticles in aqueous solution is one of the prerequisites for *in vivo* bioapplications. As shown in Figure 1E, the temporal evolutions of the D_h_ and zeta potential reveal that, the current nanoparticles can remain stable in water for at least one week. It should be mentioned that the weak positive surface potential of particles might be attributed to the unsaturated bonded Fe^3+^. The satisfying colloidal stability provides the prerequisite for the biomedical applications of the nanoparticles.

### Relaxivity and Biosafety Evaluation of Fe_3_O_4_ Nanoparticles

As we know that MRI works based on computer-assisted imaging of relaxation signals of proton spins within the human body excited by radiofrequency waves in a strong magnetic field (Wang et al., 2018). And the relaxation of proton spins to their equilibrium states via two processes, namely longitudinal relaxation, characterization by a relaxation time *T*_1_, and transverse relaxation, characterized by a relaxation time *T*_2_. The MRI contrast agents principally work by shortening the *T*_1_ or *T*_2_ relaxation times of protons located nearby. The Fe_3_O_4_ nanoparticles like what we used in the experiment, can effectively reduce the *T*_2_ relaxation time and consequently produced negative enhancement effects on *T*_2_-weighted images.

**FIGURE 2:**
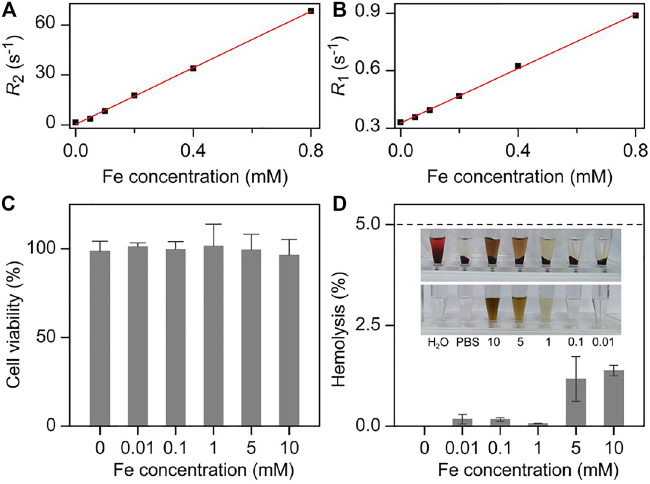
Fe concentration dependent **(A)** *R*_2_ and **(B)** *R*_1_ together with the corresponding linear fittings for extracting the molar relaxivities of Fe_3_O_4_ nanoparticles, respectively. **(C)** Viabilities 3T3 cells treated with nanoparticles at different concentrations. **(D)** The hemolysis rate analysis of Fe_3_O_4_ with different concentrations. Inset: the photographs of different solutions containing blood cells after centrifugation **(top)**, together with the nanoparticles PBS solutions with corresponding concentration as the absorbance backgrounds **(bottom)**.

Therefore, the MRI performance of Fe_3_O_4_ nanoparticles was evaluated on a 7T MRI scanner. As shown in the Supplementary Figure S4, Fe_3_O_4_ nanoparticles exhibited stronger *T*_2_ contrast enhancement effect with the increased concentration of Fe^3+^, which showed darker color in the *T*_2_-weighted imaging. By linear regression fitting of the experimental data, the transverse molar relaxivity *r*_2_ of nanoparticles was extracted as 85.0 mM^−1^ s^−1^ (Figure 2A). On the other hand, the *T*_1_ contrast enhancement ability of Fe_3_O_4_ nanoparticles was also measured. As shown in Figure 2B and Supplementary Figure S5, the longitudinal molar relaxivity *r*_1_ of nanoparticles was 0.71 mM^−1^ s^−1^ according to the slope of regression curve. Therefore, the *r*_2_/*r*_1_ ratio of the particles can be calculated as high as 119.7. The high *r*_2_ as well as high *r*_2_/*r*_1_ ratio makes them an ideal candidate for *T*_2_ contrast agents.

Any ideal contrast agent should be non- or low-toxicity to normal tissues. Therefore, the cytotoxicity of the current nanoparticles was evaluated through Cell Counting Kit-8 (CCK-8) cell proliferation assay on NIH 3T3 mouse embryonic fibroblast cells (Figure 2C). As a result, the Fe_3_O_4_ nanoparticles presented a comparable cell security, which did not show significant cytotoxicity when Fe^3+^ concentration reach 1 mM that was five orders of magnitude higher than the dose adopted for the following *in vivo* studies (10 mg/kg body weight). This lower cytotoxicity could partly be attributed to the satisfied biocompatibility of the current nanoparticles, which further highlighted the high biosafety of them.

Apart from the cytotoxicity of the nanoparticles, the hemolysis rate was investigated before the following *in vivo* experiments. As shown in Figure 2D, the pure water and the 1 × PBS solutions were set as the positive control and negative controls with 100 and 0% hemolysis rate, respectively. With respect to the Fe_3_O_4_ nanoparticles 1 × PBS solution, the calculated hemolysis rates were less than 1% at the Fe concentration of particles ranging from 0.01 to 10 mM, which significantly lower than the threshold that the hemolysis rate should be lower than 5% specified in the International Organization for Standardization (ISO) and the American Society for Testing and Materials (ASTM) (Rondon et al., 2020). These results further showed the biosafety and the blood compatibility of Fe_3_O_4_ nanoparticles in the blood stream, providing a prerequisite for the intravenous injection of the nanoparticles.

### Visualization of the Potential Central Nervous System Impairment Caused by PMB

**FIGURE 3:**
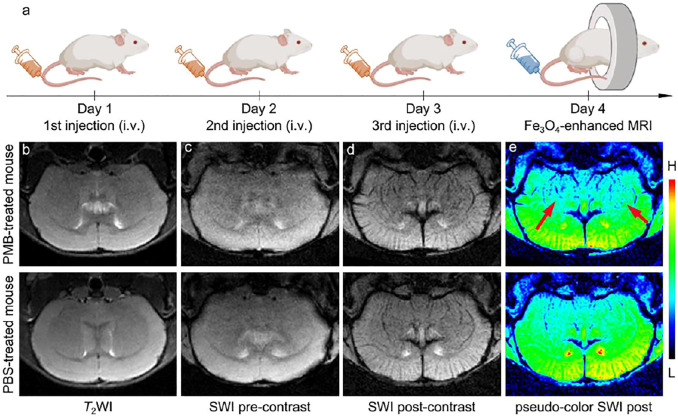
**(a)** Schematic illustration of injection procedure of the PBS or PMB, the nanoparticles administration modalities, and brain MR imaging. **(b)** *T*_2_-weighted imaging of the brain of PBS- or PMB-treated mice, respectively, before injecting the Fe_3_O_4_ nanoparticles. SWI of the brain of PBS- or PMB-treated mice, respectively, before **(c)** and after **(d)** injecting the Fe_3_O_4_ nanoparticles, together with the SW images in **(d)** with pseudocolor **(e)**.

On the basis of the outstanding MRI performance and biosafety of the Fe_3_O_4_ nanoparticles in the *in vitro* experiments, it was employed to visualize the central nervous system damage caused by PMB *in vivo*. Six BALB/c mice were randomly divided into two groups (*n* = 3), and were intravenously (i.v.) injected with PMB (6 mg per kg weight) for one group or the same volume of 1 × PBS for anther group once a day for three days, and then one mouse from each group was used as a representative and subjected to the MR imaging, as shown in Figure 3A. Before injection of Fe_3_O_4_ nanoparticles, *T*_2_-weighted MR images of brain section was obtained and provided in the Figure 3B. It seems that the brain anatomic structures of mice injected with either PBS or PMB did not show significant damage.

In the previous studies, MR susceptibility-weighted imaging (SWI) is a high-resolution and 3D gradient-echo *T*_2_* MR technique, which is very sensitive to the magnetic susceptibility difference, particularly suitable for blood, hemorrhage, and iron storage sensitive imaging. Therefore, for clearly visualize the potential central nervous system damages caused by PMB injection, the brains of mice were further studied with a susceptibility-weighted imaging (SWI) sequence with the help of superparamagnetic Fe_3_O_4_ nanoparticles. The susceptibility-weighted images were acquired pre- and post-injection of the Fe_3_O_4_ nanoparticles. The nanoparticle–enhanced SWI were shown in Figures 3C,D, after the intravenous administration, the contrast of the cerebral vessels in both PMB- and PBS-treated mice were strongly enhanced. However, in comparison with the PBS-treated mouse, the nanoparticles extravasated into the brain parenchyma in the PMB-treated mouse, which resulted in a darker color surrounding the striatum in the image, identifying the potential BBB damaged sites. To further highlight these SWI signals, the post-contrast SWI was handled by pseudo-color processing, as shown in Figure 3E. Contrasting to the PBS-treated mouse, several discrete punctate signals distributed asymmetrically across brain regions of PMB-treated mouse, as indicated by the red arrows, which suggested that the structure of lenticulostriate artery may have been impaired by continuous administration of PMB.

**FIGURE 4:**
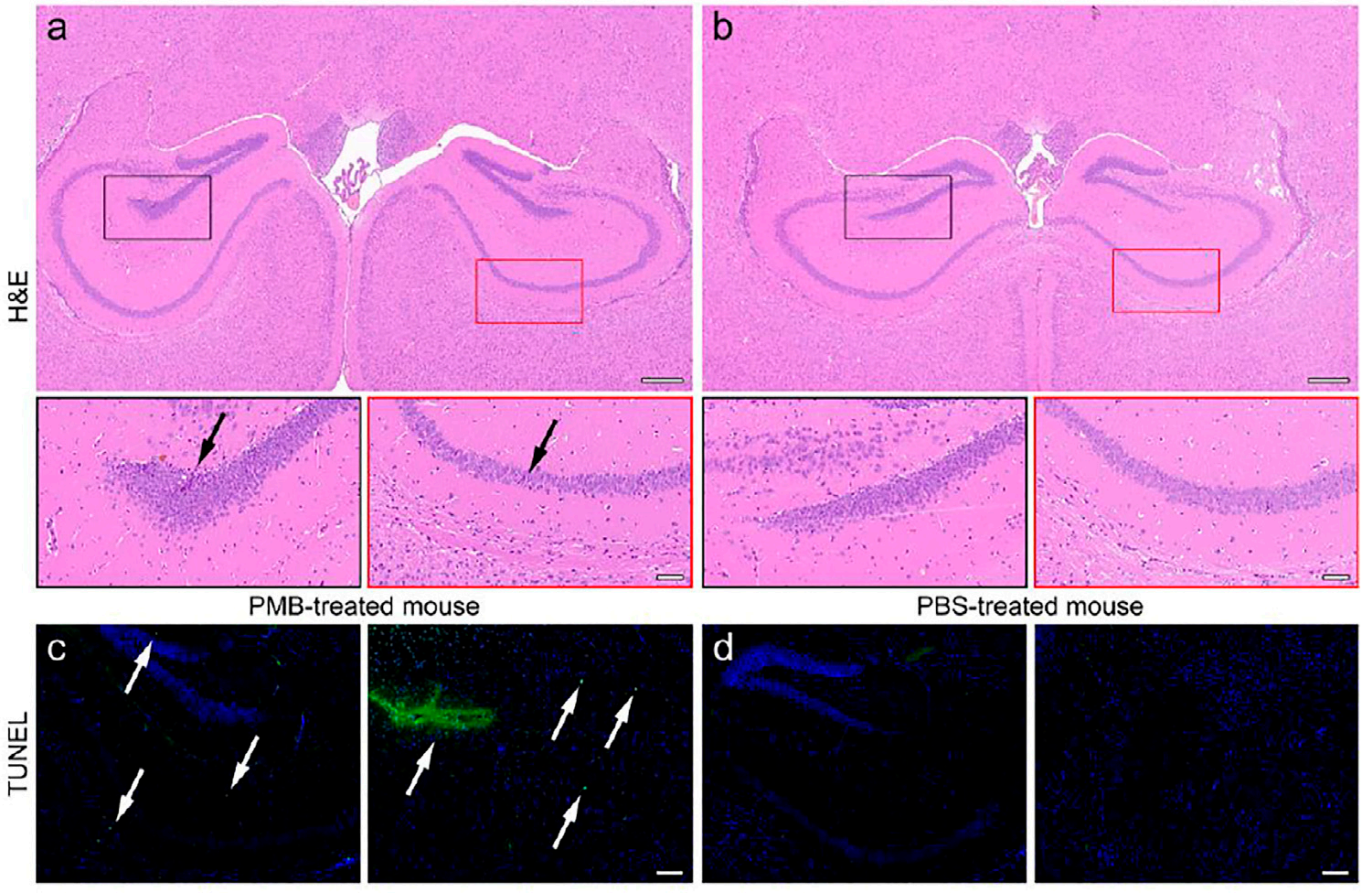
H&E staining of the brain slices of **(a)** PMB-treated mouse and **(b)** PBS-treated mouse, in which the DG and CA1 region in black and red frames are enlarged in the bottom, respectively. TUNEL staining of the brain slices of **(a)** PMB-treated mouse and **(b)** PBS-treated mouse, where the positive sites are highlighted by white arrows. The embedded scale bar in the top, the bottom of **(a,b)**, and **(c,d)** corresponds to 100, 50, and 100 μm, respectively.

For further confirming the pathological changes in the brains, the PMB- and PBS-treated mice were sacrificed after SWI. The brain tissues of them were extracted, cut into slices, and subjected to hematoxylin-eosin (H&E) staining for the histopathological analysis. The representative field of view of slices are shown in Figures 4A,B. Accordingly, in the brain slice of PMB-treated mouse, several neurons in dentate gyrus (DG) region (black frame) and cornu ammonus area 1 (CA1) (red frame) were atrophied with deeper stained color, and the cytoplasm and nucleus of them were poorly demarcated, as indicated by the black arrows. In contrast, the neurons in the same regions of PBS-treated mice were arranged regularly with the normal morphological structure. The boundary between cytoplasm and nucleus of the cells can be clearly differentiated. This PMB-caused brain impairment can also be quantitatively characterized by the abnormal cells in the hippocampal region of brain slices (Supplementary Figure S6).

In addition, the terminal deoxynucleotidyl transferase (TdT) mediated dUTP Nick End Labeling (TUNEL) assay was also carried out to check whether the nervous cells underwent extensive DNA degradation, which is the striking feature of apoptosis. Almost no nuclei were stained by TUNEL in the brain of PBS-treated mice. However, the TUNEL-positive cells were appeared surrounding the CA1, CA2, and DG regions of the brain extracted from PMB-treat mouse, shown in the left panel of Figures 4C,D, respectively, indicating that PMB may impair the activity of neurons within the hippocampus of the brain. The quantitative statistical results of TUNEL-positive cells in hippocampus also confirmed this conclusion (Supplementary Figure S7). Moreover, the blood vessels surrounding the caudate putamen (CPu) area of the brain were impaired in the PMB-treated mice. This result suggested that PMB may lead to the destruction of the neurons and BBB in brain, which is consistent with the imaging results in Figure 3, indicating that the Fe_3_O_4_ enhanced SWI in the current work is precise and reliable.

## Conclusion

In summary, the biocompatible ultra-uniform Fe_3_O_4_ nanoparticles were prepared for noninvasively *in vivo* visualizing the potential impairment of PMB to the central nervous system. Systematic studies combining both *in vitro* and *in vivo* experimental results clearly reveal that Fe_3_O_4_ nanoparticles can serve as an appropriate contrast agent with high transverse relaxivity and outstanding biosafety, which thus enables the following SWI studies on the PMB-treated mice models to mimic the clinical PMB therapies. Through Fe_3_O_4_ nanoparticle enhanced SWI, it is first found that the BBB of mice may be impaired by successive PMB administration, displaying by the discrete punctate SWI signals distributed asymmetrically across brain regions in brain parenchyma.

In brief, the current studies provide an effective strategy for visualizing the impairment of PMB to central nervous system *in vivo*, which may pave a noninvasive approach for in-depth studies of PMB medication approach, such as individually evaluating the dosage and administration frequency of PMB among different patients, monitoring the BBB changes during treatment, and even assessing the risk after BBB successive medication in multidrug-resistant Gram-negative bacterial infected patients from the perspective of medical imaging.

## Experimental Sections

### Chemicals

Ferric trichloride hexahydrate (FeCl_3_•6H_2_O, 99.0%) was purchased from Shandong Xiya Chemical Industry Company (Shandong, China). OA and NaOH were purchased from Sigma-Aldrich. Tetrahydrofuran (THF) was purchased from Fuchen (Tianjin) Chemical Reagent, Co., Ltd (Tianjin, China). Cyclohexane was purchased from Tianjin Damao Chemical Reagent Factory (Tianjin, China). Analytical grade chemicals such as acetone and 1-octadecene were purchased from Beijing Chemical Reagent, Co., Ltd (Beijing, China). All the above chemicals were used without further purification. Polymyxin B (≥6,000 u/mg) sulfate was purchased from Beijing Solarbio Science & Technology Co., Ltd. The BALB/c mice were purchased from Beijing Vital River Laboratories (Beijing, China). The sodium pentobarbital was purchased from Baxter Healthcare Corporation.

### Synthesis of OA-Capped Fe_3_O_4_ Nanoparticles

OA-capped Fe_3_O_4_ nanoparticles were synthesized through thermal decomposition method. In brief, 3.6 g (4 mmol) of prepared iron oleate and 3.39 g (4 mmol) of OA were dissolved in 25 ml of 1-octadecene. The resultant mixture was heated to 310°C with a rate of 3.3°C min^−1^, and then maintained at 310°C for 30 min under nitrogen protection. The preparation was terminated by cooling the reaction mixture down to room temperature. The resultant OA-capped Fe_3_O_4_ nanoparticles were precipitated by acetone, collected by magnetic separation, washed with acetone several times, and finally re-dispersed in THF or cyclohexane for further experiments.

### Ligand Exchange

Typically, 20 mg of dp-PEG-OCH_3_ was dissolved in 5 ml of THF containing 2 mg OA-capped Fe_3_O_4_ nanoparticles. Then, the reaction mixture was heated to 40°C and kept at this temperature overnight under stirring. After that, the resultant NP-PEG nanoparticles were precipitated by cyclohexane, washed with cyclohexane for three times, and then dried under vacuum at room temperature. To remove the excess free PEG ligand, the NP-PEG nanoparticles dissolved in MilliQ water were further purified through ultrafiltration with 30 kDa MWCO centrifugal filter (Millipore YM-100) for 5 cycles at 3,000 g.

### Characterizations of Fe_3_O_4_ Nanoparticles

TEM images of the nanoparticles were taken on a JEM-2100 transmission electron microscope at an acceleration voltage of 200 kV. The particle size was determined by averaging at least 25 nanoparticles per sample. DLS measurements were carried out at 298 K with Nano ZS (Malvern) equipped with a solid state He-Ne laser (λ = 632.8 nm) for determining the hydrodynamic size of the different particles. The iron contents in different systems were determined with 1,10-phenanthroline through absorption spectroscopy after the particles were eroded with concentrated hydrochloric acid. The absorption of water solution of nanoparticles was also analyzed through absorption spectroscopy at room temperature on the UV-Vis spectrophotometer (Thermo, MULTISKAN GO).

### Relaxivity Measurements

The relaxivity measurements were carried out on a 7.0 T Bruker Biospec animal MRI instrument (BioSpec70/20USR). A series of aqueous solutions containing nanoparticles with different Fe concentration were prepared in 200 μl Eppendorf tube for MR studies. The detailed parameters for *T*_1_ and *T*_2_ measurements were set as follows: TE = 5.01 ms, TR = 300 ms, MTX = 200 × 200, and FOV = 40 × 40 mm^2^ for *T*_1_-weighted imaging; TE = 40 ms, TR = 3,000 ms, MTX = 200 × 200, and FOV = 40 × 40 mm^2^ for *T*_2_-weighted imaging.

### Cell Culture

Mouse embryonic fibroblast cell line NIH 3T3 was cultured in a medium of Dulbecco’s Modified Eagle Medium (DMEM) high glucose and F-12K nutrient mixture (1: 1) supplemented with 10% fetal bovine serum and 1% penicillin–streptomycin solution (100×) at 37°C under a 5% CO_2_ atmosphere.

### Cytotoxicity of Nanoparticles

Cell viability was determined by CCK-8 assay. 3T3 Cells were seeded into a 96-well cell culture plate by 5 × 10^3^ per well under 100% humidity, and then cultured at 37°C for 24 h. The Fe_3_O_4_ nanoparticles with a series of Fe^3+^ concentration were added to the wells respectively and incubated with the cells for another 24 h at 37°C. Subsequently, the supernatants containing the free formulations were decanted. CCK-8 (100 μl, 10 mg/ml) was added to each well and incubated for 3 h at 37°C. The optical density of each well at 450 nm was recorded on a microplate reader (Thermo, Multiskan GO).

### Hemolysis Test

Hemolysis test was conducted according to the previous report (Zhang et al., 2007; Love et al., 2012). Briefly, a 2 ml blood sample was mixed with 6 ml of phosphate-buffered saline (PBS) and centrifuged at 1,500 rpm for 15 min. The supernatant was drawn off and five subsequent washes were carried out before diluting the sample to a final volume of 1 ml in PBS. The red blood cells were then diluted 1: 4 into 1 × PBS solutions (negative control); water (positive control); nanoparticle solutions (in PBS) at varying concentrations (tested samples).

Due to the broad absorption of nanoparticles, the absorption background was composed of PBS containing the nanoparticle at corresponding concentrations without red blood cells. The samples were left at room temperature in the dark for 4 h at 37°C and then centrifuged at 3,000 rpm for 5 min. After centrifuging, the supernatant was transferred to a 96-well plate and the absorbance at 541 nm was recorded.

The hemolysis rate was calculated by the following equation:

$$\mathrm{Hemolysis rate}=\;\frac{D_{t}-D_{b}}{D_{\mathrm{pc}}-D_{\mathrm{nc}}}\times 100\%$$

where D_t_, D_b_, D_nc_, and D_pc_ were the absorbance of the tested sample, the background sample, the negative control and the positive control, respectively.

### Animal Models

6-week-old male BALB/c mice were used in this study. All mice were maintained in ventilated cages at a temperature of 20–25°C and a relative humidity of 30–50% under a 12 h light/dark cycle and were given free access to food and water.

Three mice were intravenously injected with PBS solution of PMB (1 mg/ml) at a dosage of 6 mg per kg weight once a day for 3 days, while the other three mice were intravenously injected with pure PBS solution with the same volume. The mice were subjected to MR scanning for brain imaging in the 4^th^ day.

### *In Vivo* MRI Assessment

The brain MR imaging was carried out on a 7.0 T MRI system (Bruker BioSpec70/20 USR) equipped with a rat head surface coil to receive signals. The MR images pre- and post-contrasted with nanoparticles were acquired using a gradient echo SWI sequence. The imaging parameters are set as follows: repetition time (TR) = 469.8 ms, echo time (TE) = 8.07 ms, field of view = 20 × 20 mm, matrix = 200 × 200, and slice thickness = 1 mm *T*_2_WI sequence were also obtained pre-contrast. The detailed parameters for *T*_2_WI were set as follows: TR = 2,366 ms, TE = 35 ms, field of view = 20 × 20 mm, and matrix = 200 × 200.

During the MRI experiments, the animals were anesthetized with 2% isoflurane in oxygen-mixed air via a facemask. Rectal temperature was maintained at 37 ± 1°C. The nanoparticles were injected into the tail vain at the injection dose of 10 mg Fe per kg body weight, and the final concentration of Fe was 1.5 mg Fe mL^−1^ in PBS.

### Histopathological and Immunohistochemistry Analysis

After MR imaging, the mice were sacrificed, and the brain tissues were extracted, fixed with formalin, and embedded in paraffin. After that, the brain tissues were sliced with an ultrathin semiautomatic microtome to obtain coronal sections of 3 µm. The adjacent slices were selected for H&E and TUNEL staining, respectively.

All animal experiments reported herein were performed according to a protocol approved by the Peking University Institutional Animal Care and Use Committee.

## Data Availability

The original contributions presented in the study are included in the article/Supplementary Material, further inquiries can be directed to the corresponding authors.
