# Supplementary material for: Visualizing the Potential Impairment of Polymyxin B to Central Nervous System Through MR Susceptibility-Weighted Imaging
Source: Front Pharmacol. 2021 Dec 2;12:784864. doi: 10.3389/fphar.2021.784864 (PMC8675099; doi:10.3389/fphar.2021.784864)
Supplement: Supplementary file 1 [file DataSheet1.doc]

Supplementary Material

# Supplementary Data

## Supplementary Figures


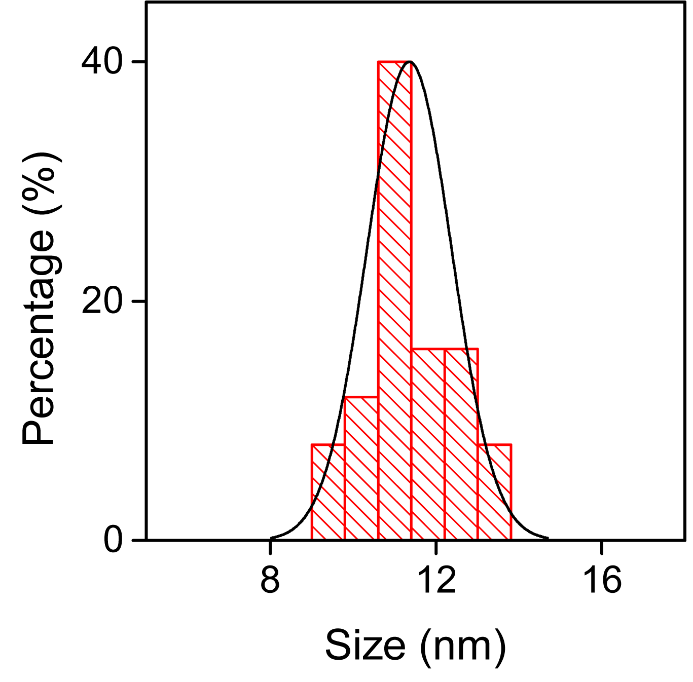


**Supplementary Figure 1.** The histogram showing the size distribution profile of OA-capped Fe_3_O_4_ nanoparticles.


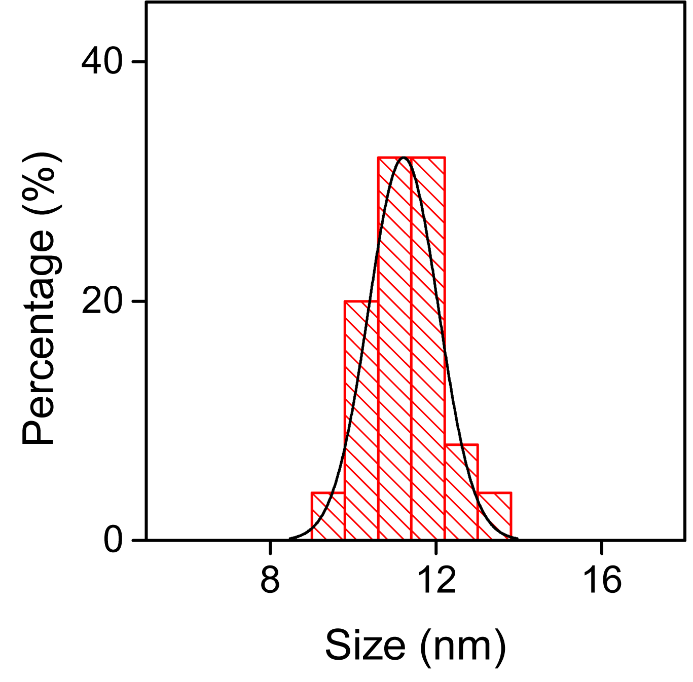


**Supplementary Figure 2.** The histogram showing the size distribution profile of biocompatible Fe_3_O_4_ nanoparticles.


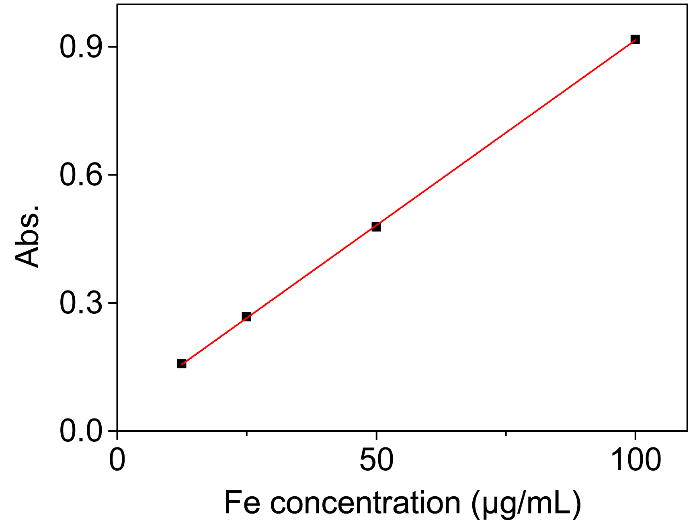


**Supplementary Figure 3.** The linear correlation between 400 nm absorbance and concentration of Fe in the aqueous solution of nanoparticles for showing the compliance with Beer-Lambert law.


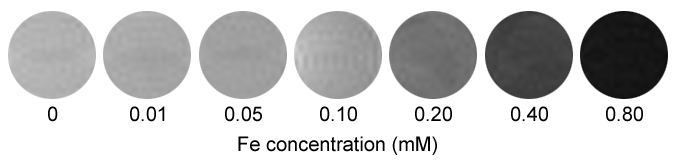


**Supplementary Figure 4.** *T*_2_-weighted MR images of a series of nanoparticle solutions.


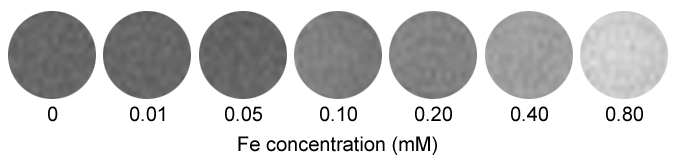


**Supplementary Figure 5.** *T*_1_-weighted MR images of a series of nanoparticle solutions.


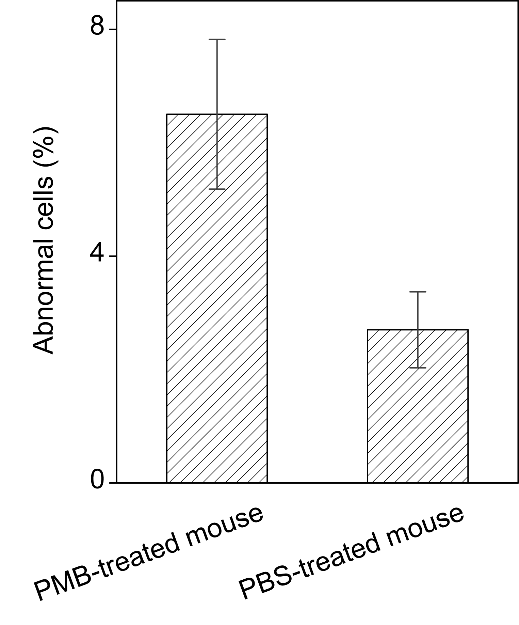


**Supplementary Figure 6.** Percentage of abnormal cells in the hippocampal region of brain slices.


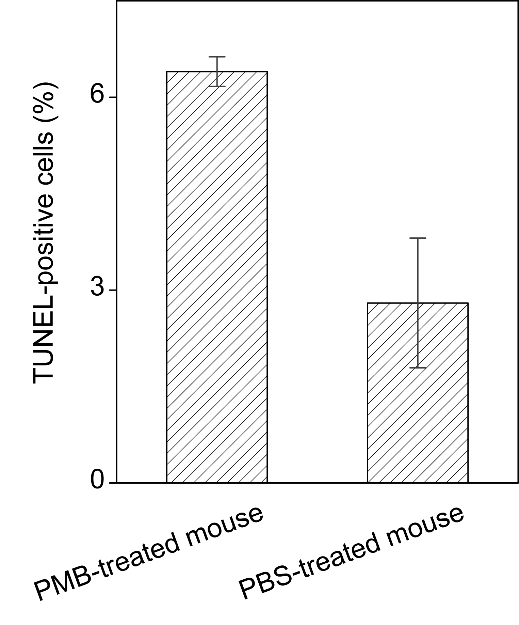


**Supplementary Figure 7.** Percentage of TUNEL-positive cells in the hippocampal region of brain slices.
